# Pigment-dispersing factor is present in circadian clock neurons of pea aphids and may mediate photoperiodic signalling to insulin-producing cells

**DOI:** 10.1098/rsob.230090

**Published:** 2023-06-28

**Authors:** Francesca Sara Colizzi, Jan A. Veenstra, Gustavo L. Rezende, Charlotte Helfrich-Förster, David Martínez-Torres

**Affiliations:** ^1^ Neurobiology and Genetics, University of Würzburg, Biocenter, Am Hubland, 97074 Würzburg, Germany; ^2^ Université de Bordeaux, INCIA CNRS UMR, 5287 Talence, France; ^3^ Universitat de València, Institut de Biologia Integrativa de Sistemes, Parc Cientific, C/ Catedrático Agustín Escardino Benlloch no. 9, 46980 Paterna, València, Spain

**Keywords:** pigment-dispersing factor, pea aphid, photoperiod, clock neurons, insulin-like peptides, Cryptochrome

## Abstract

The neuropeptide pigment-dispersing factor (PDF) plays a pivotal role in the circadian clock of most Ecdysozoa and is additionally involved in the timing of seasonal responses of several photoperiodic species. The pea aphid, *Acyrthosiphon pisum,* is a paradigmatic photoperiodic species with an annual life cycle tightly coupled to the seasonal changes in day length. Nevertheless, PDF could not be identified in *A. pisum* so far*.* In the present study, we identified a PDF-coding gene that has undergone significant changes in the otherwise highly conserved insect C-terminal amino acid sequence. A newly generated aphid-specific PDF antibody stained four neurons in each hemisphere of the aphid brain that co-express the clock protein Period and have projections to the *pars lateralis* that are highly plastic and change their appearance in a daily and seasonal manner, resembling those of the fruit fly PDF neurons. Most intriguingly, the PDF terminals overlap with dendrites of the insulin-like peptide (ILP) positive neurosecretory cells in the *pars intercerebralis* and with putative terminals of Cryptochrome (CRY) positive clock neurons. Since ILP has been previously shown to be crucial for seasonal adaptations and CRY might serve as a circadian photoreceptor vital for measuring day length, our results suggest that PDF plays a critical role in aphid seasonal timing.

## Graphical abstract

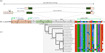

## 1. Introduction

Photoperiodism is the ability to perceive day length (photoperiod) as an anticipatory cue of seasonal changes and to respond with appropriate physiological and behavioural adjustments [1]. A well-known example of a photoperiodic response is the induction of overwintering diapause in many insect species in response to day length shortening that announces the arrival of the winter [2]. Aphids (Hemiptera: Aphididae) were the first animals described as photoperiodic [3] and constitute a good model to study how a photoperiodic system is built. They have a remarkable life cycle, during which winter-resistant diapausing eggs are produced by sexual reproduction, when photoperiod shortens in autumn. During spring and summer, characterized by longer days, aphid populations consist exclusively of viviparous females that reproduce parthenogenetically (i.e. they are clonal) for several generations. New embryos are already developing inside the yet unborn older embryos developing inside parthenogenetic aphids, which ensures rapid and efficient reproduction. When autumn comes and the days shorten, aphids switch their reproductive mode and generate a single generation of males and oviparous sexual females, which mate and produce cold-resistant diapausing eggs that overwinter and survive the unfavourable season. In the next spring, those eggs hatch and the newly born nymphs initiate a new series of viviparous parthenogenetic female generations that succeed one another until the next autumn. This strategy ensures survival over winter and increases genetic variability and the chance that some of the young aphids hatching next spring will be optimally adapted to the as yet unknown new conditions [4].

Thus, the ability to measure day length is essential for insect (and aphid) survival in temperate regions of the planet. Measuring day length requires an endogenous timing system as a reference, and the circadian clock is hypothesized to fulfil this role [5]. The circadian clock would then be an essential component of a complex photoperiodic system, in which dedicated photoperiodic photoreceptors and effector molecules also play a role, distinguishing day from night and relaying the photoperiodic message to achieve the corresponding outcome [2,6]. However, knowledge on the molecular and cellular basis of the photoperiodic system in insects is currently fragmentary. Here, we investigate the putative role of the neuropeptide pigment-dispersing factor (PDF) in the photoperiodic system of aphids.

PDF plays a pivotal role in the circadian clock of most insects investigated so far [7–10]. In fruit flies and cockroaches, PDF is an essential output molecule of specific circadian clock neurons that control behavioural rhythmicity and serves additionally as a communication factor within the circadian clock network [9,11–13]. In addition, what is most relevant for the present report is that, in several insects, including fruit flies, PDF is implicated in seasonal timing [14–20] and, in one moth species, in annual rhythms [21]. In the blow fly *Protophormia terraenovae*, the ablation of the PDF-positive clock neurons renders the flies unable to discriminate between long days (LDs) and short days (SDs) [14] and thus unable to prepare in time for the coming winter. In the bugs *Plautia stali* [18] and *Pyrrhocoris apterus* [19], PDF is essential for entering overwintering diapause under SDs, while in female mosquitoes (*Culex pipiens*) [15] and fruit flies [17], PDF is necessary to maintain the ability to reproduce during long summer days. Furthermore, fruit flies and cockroaches (*Rhyparobia maderae*) have higher PDF levels/denser PDF branching patterns under long summer days than under short winter days [20,22]. All these findings suggest that PDF conveys day length information to the photoperiodic system: in some insects, PDF appears to signal LDs, while in others it rather signals SDs. Besides all the above, PDF has also been implicated in many other functions such as negative geotaxis [23], long-term memory [24,25], pheromone production [26], renal physiology [27] and general metabolism [28] of fruit flies.

However, despite the remarkable ability of aphids to measure day length, they seemed to lack PDF. Neither the *pdf* gene [29] nor the PDF peptide [30] have been detected in aphids to date, and we hypothesized that the lack of this neuropeptide could be a possible cause for the weak activity rhythms of aphids that dampen quickly after transfer into constant darkness [31]. However, other species of the order Hemiptera, such as the cicada *Meimuna opalifera* [32] and different species of bugs, such as *Gerris paludum*, *Rhodnius prolixus* and *Riptortus pedestris* [33–35], do possess PDF. Some of these species are strongly photoperiodic, and as mentioned above, PDF appears to be involved in their photoperiodic responses.

Since identifying orthologous short neuropeptides may become very difficult when amino acid sequences are poorly conserved, it could not be ruled out that aphids indeed possess PDF but that its sequence diverged so much during evolution that neither similarity-based BLAST searches, nor antibodies recognizing PDFs in other insects were able to identify it. Furthermore, the aphid PDF putative receptor was successfully identified [36,37]. Such was the case for the beetle *Tribolium castaneum*, for which initially no PDF gene sequence could be identified [38], while the G protein-coupled PDF receptor was immediately detected [39]. Eventually, a comparative analysis of several beetle genome sequences and RNAseq assemblies revealed the PDF sequence in *T. castaneum* and that it had undergone significant changes, especially in the C-terminal amino acid sequence [39]. Similarly, thanks to the current availability of genome and transcriptome assemblies for diverse aphid and aphid-related species, we finally report here the finding of a highly divergent *pdf* gene in the pea aphid genome that was already present in the ancestor of all the Aphidomorpha.

Using a newly generated antibody against the predicted amino acid sequence of *A. pisum* precursor PDF, we were able to stain four neurons in each lateral protocerebrum of the aphid brain that co-express the clock protein Period and show projections into the superior protocerebrum, strongly resembling those of the fruit fly PDF neurons. They overlap with fibres of Cryptochrome (CRY) positive clock neurons in the lateral and superior protocerebrum and with the dendrites from the insulin-like peptide (ILP) positive neurosecretory cells in the *pars intercerebralis*. The PDF terminals in the superior protocerebrum are highly plastic and change their length in a daily and seasonal manner. Together, our results establish the presence of PDF in aphids and suggest that PDF plays a pivotal role in the circadian and seasonal timing of pea aphids, which represents a significant advance in the field of seasonal biology in aphids and insects in general.

## 2. Results

### 2.1. Pea aphids possess a highly divergent pigment-dispersing factor-encoding gene

As typical for neuropeptides, PDF is synthesized from a larger, inactive precursor protein (prepro-PDF), which consists of a signal peptide and a PDF-associated peptide (PAP) followed by the region that, after processing, will constitute the mature PDF [40]. The signal peptide guides the protein to the secretory pathway and is later cleaved off. Similarly, the mature PDF is cut out from the PAP by neuropeptide convertases that recognize single or paired basic residues as cleavage sites [41] rendering a peptide consisting of 18 amino acids highly conserved across Arthropoda [40]. Finally, the neuropeptide is further processed and amidated at its C-terminal end to become biologically active [42].

**Figure 1.**
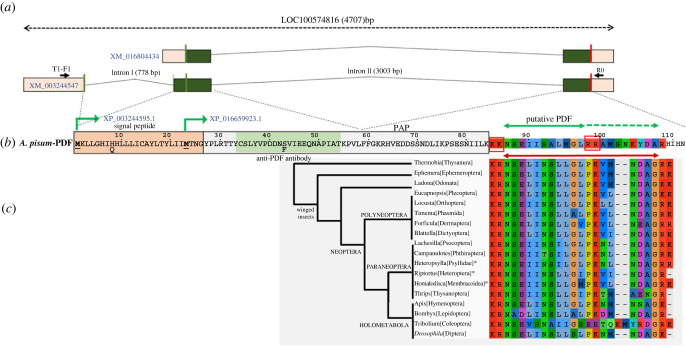
Identification and characterization of PDF in *A. pisum*. (*a*) Schematic representation of the two predicted transcripts (indicated by accession numbers in blue) that encode predicted proteins that partially aligned with query sequences in BlastP searches. Predicted exons are indicated in coloured boxes. Dark green in exons correspond to CDSs. Vertical green lines correspond to initial methionines and vertical red lines to stop codons. Black lines connecting exons indicate predicted introns (size indicated in parentheses). The position of the primers used to experimentally characterize the transcripts is indicated by black arrows. (*b*) Predicted protein isoforms (indicated by their accession numbers in blue) from the two predicted transcripts in (*a*). Both proteins start at different methionines (green angled arrows). Dotted lines connect regions of the protein encoded by CDSs in particular exons. Different elements of the PDF precursor are indicated: signal peptide, PAP (PDF-associated propeptide). The carboxyl-terminal region that partially aligns with insect PDF appears in background colours. A putative mature PDF peptide (see §§2.1, 2.2 and electronic supplementary material, figure S1) is indicated by a double green arrow limited by red boxes that correspond to predicted convertase cleavage sites. Dotted green arrow indicates a possible extension of a hypothetical mature peptide if the second convertase cleavage site is not cleaved. Two polymorphisms found in different strains are indicated below the main predicted sequence. The green shadowed sequence indicates the peptide used to raise the pea aphid-specific antiserum. (*c*) PDF sequences, along with flanking basic residues, from 18 insect species representative of major insect orders, aligned with the *A. pisum* predicted peptide. The double red arrow indicates the extension of the mature PDF in these insects (note the highly divergent sequence of *Tribolium*). A manually built cladogram beside the alignment shows the relationships between insect groups according to [44] (only the genus and order are indicated). Asterisks (*) indicate three representatives from Hemiptera. Close to some branches, in capital letters, main insect groupings are indicated. NCBI accession numbers for the included sequences are, from top to bottom: JT495639, GAUK02023238, KAG8239166, GIEC01052504, AKN21252, CAD7590987, GAYQ02044840, PSN52637, GCWJ01020925, GCWD01026746, GCXB01024081, BAN82692, XP_046677079, XP_034243662, XP_006570344, NP_001036920, EFA10486, O96690.

Our tblastn searches for pea aphid PDF on the genome assembly yielded a small protein sequence that had rather limited sequence similarity to PDF. Using this sequence as a query in a tblastn search on transcriptome shotgun assemblies of Aphidomorpha identified a number of aphid transcripts. The putative aphid PDF sequences in these transcripts are conserved and they all start with a signal peptide. This strongly suggested that these are the aphid PDF precursors. Our BlastP searches with these putative PDF precursors using the NCBI refseq_protein database restricted to *A. pisum* (AL4 genome assembly [43]) (see Material and methods) yielded two hits. These corresponded to two predicted proteins (XP_003244595.1 and XP_016659923.1) described as ‘uncharacterized protein LOC100574816’ isoforms X1 and X2, respectively, of which the first one is an orthologue of the putative PDF precursors (figure 1). The N-terminal convertase Lys-Lys-Lys cleavage site flanking the sequence of the putative mature PDF in the predicted proteins (figure 1) is very unusual [41], while the internal Arg-Arg cleavage site, if cleaved, would yield a very short PDF (11 amino acids) or, if left intact, the C-terminal end of the molecule would be very different from other insects (figure 1). Since none of these hypothetical cleavages would result in a credible PDF neuropeptide, the partial sequence similarity with PDF found in previous attempts to identify this gene [29] was interpreted as coincidental rather than genuine. The presence of orthologous transcripts in other aphid species, and even the more distantly related grape phylloxera *Daktulosphaira vitifoliae*, showed the putative neuropeptide to be conserved and, hence, likely functional (see electronic supplementary material, figure S1).

The two pea aphid predicted proteins correspond to conceptual translations from two alternative predicted transcript variants, starting at different sites, of gene LOC100574816 that spans over 4707 bp (figure 1). Predicted transcript XM_003244547 spans 926 nucleotides (nts) distributed in three exons (figure 1*a*). This mRNA is predicted to contain a 339 nts coding sequence (CDS) the initial ATG codon of which is distributed between the end of the first exon (AT) and the beginning of the second one (G) (figure 1*a*,*b*). Thus, this mRNA can be translated into predicted protein XP_003244595.1 consisting of 112 amino acids (figure 1*b*). Adding further evidence to this gene encoding a true pea aphid PDF, this protein contains the signal peptide (as predicted by SignalP 6.0 [45]) typical of PDF precursor proteins, apart from the two protein convertase cleavage sites discussed above, necessary to get the mature PDF from the propeptide (figure 1*b*).

The second predicted transcript (XM_016804434) would yield the predicted protein isoform XP_016659923.1, which is identical in all its length to protein XP_003244595.1 but lacking most of the predicted signal peptide (figure 1*a*). Given its questionable functionality, it might likely correspond to an artefactual prediction. In fact, this transcript is an automatic prediction based on only a few RNAseq reads. We thus excluded this transcript from further analysis.

To experimentally validate the *A. pisum* putative PDF transcript predictions, we designed specific primers based on the 5′ and 3′ UTRs of the predicted transcript XM_003244547 (figure 1*a*) and used them to PCR amplify the corresponding transcripts from cDNAs synthesized using total RNA purified from different pea aphid strains (see Material and methods). Our primer combination yielded amplified fragments of the expected size according to the prediction.

We performed direct sequencing of the PCR-amplified fragments from the main transcript (XM_003244547), that encodes the full putative pre-propeptide, from seven pea aphid strains from diverse geographical origins (see Material and methods), including the LSR1 strain, whose genome was firstly published [46]. All sequences were deposited in GeneBank (accession numbers indicated in the electronic supplementary material, table S1). For all the strains, our experimental sequences coincided, for the most part, with the predicted sequence. However, we found two non-synonymous polymorphisms at amino acid positions 8 and 43, that, in the predicted transcript, correspond to histidine (H) and serine (S), respectively (figure 1*b*). We found that five out of the seven strains sequenced (including the reference strain LSR1) were in fact heterozygous at both positions having, in addition to the predicted H and S, glutamine (Q) and phenylalanine (F), respectively, at those two positions (figure 1*b*). Sequencing of the cloned sequences revealed that alleles H and S reside on the same chromosome, and Q and F on the other one. Coincident with these results, two strains were homozygous at both positions. Strain BOL was QF while strain GR was HS (electronic supplementary material, table S1). It is yet unclear as to the relevance, if any, of these polymorphisms (but see below).

Finally, our experimental sequences from the seven pea aphid strains perfectly matched the 3′ end sequence of transcript predictions that correspond to the carboxyl-terminal end of the predicted PDF propeptide containing the putative mature PDF described above. Figure 1*c* shows the alignment of this region in the pea aphid with 18 sequences representative of major insect orders. This region in the pea aphid seems to have diverged much when compared with other insect groups (with the exception of *Tribolium*, which also possesses a rather divergent PDF; figure 1*c*) especially after the second putative convertase cleavage site. Indeed, for the 25 amino acid positions aligned in figure 1*c*, the average number of differences among the 17 insect species (excluding both *Tribolium* and *A. pisum*) is 4.3 (ranging from 1 to 9). However, the average number of differences between the *A. pisum* sequence and the rest is 13.1 (ranging from 12 to 15).

### 2.2. A divergent pigment-dispersing factor is characteristic of Aphidomorpha

To investigate whether the putative PDF found in Blast searches in the pea aphid was also present in other aphid species, we performed BlastP or tblastn searches in different aphid databases using as query the pea aphid sequence identified in the above section (see Material and methods). We found highly similar sequences to the putative *A. pisum* PDF in all searched aphid databases including different species of the two tribes in the subfamily Aphidinae (i.e. Macrosiphini, to which *A. pisum* belongs, and Aphidini), representatives of Eriosomatinae and Lachninae (two distantly related subfamilies within the Aphididae [47]), and, most relevant, in representatives of oviparous families Adelgidae and Phylloxeridae, which separated from true aphids some 200 Mya [48] (see electronic supplementary material, figure S1). These results led us to conclude that we had indeed found the pea aphid PDF. Furthermore, the 11 amino acids, flanked by the two convertase cleavage sites, present in the *A. pisum* sequence, are identical in most aphid sequences including the Adelgidae and Phylloxeridae representatives (figure 1*c*; electronic supplementary material, figure S1). Interestingly, the unusual Lys-Lys cleavage site observed in the *A. pisum* sequence is also present in most other aphid species, but is replaced by conventional Lys-Arg in oviparous families Adelgidae and in the grape phylloxera *Daktulosphaira vitifoliae* and also in the single representative of the distantly related aphid subfamily Eriosomatinae (figure 1*c*; electronic supplementary material, figure S1). This degree of conservation in the sequence delimited by the two putative convertase sites points to this short peptide being the aphid PDF active form, although additional experiments should confirm this hypothesis. It is worth noting that none of the aphid PDFs can be C-amidated, as they all lack a terminal glycine (see electronic supplementary material, figure S1).

Thus, although the PDF neuropeptide is different from that of other insect species, within the Aphidomorpha it is well conserved. However, the remainder of the PDF precursor has evolved significantly in aphids. In fact, a phylogenetic tree built using the whole PDF precursor sequences (electronic supplementary material, figure S1) recovers the main aphid groups and known evolutionary relationships among them. As expected, the most divergent sequences correspond to representatives of basal families Adelgidae and Phylloxeridae, in the latter case to the point that an unambiguous signal peptide is no longer predicted by SignalP 6.0. Thus, our results point to a divergent PDF neuropeptide already present in the ancestor of all Aphidomorpha and the gene evolving in the group since then.

### 2.3. The pigment-dispersing factor antibody labels four neurons in the lateral brain

**Figure 2.**
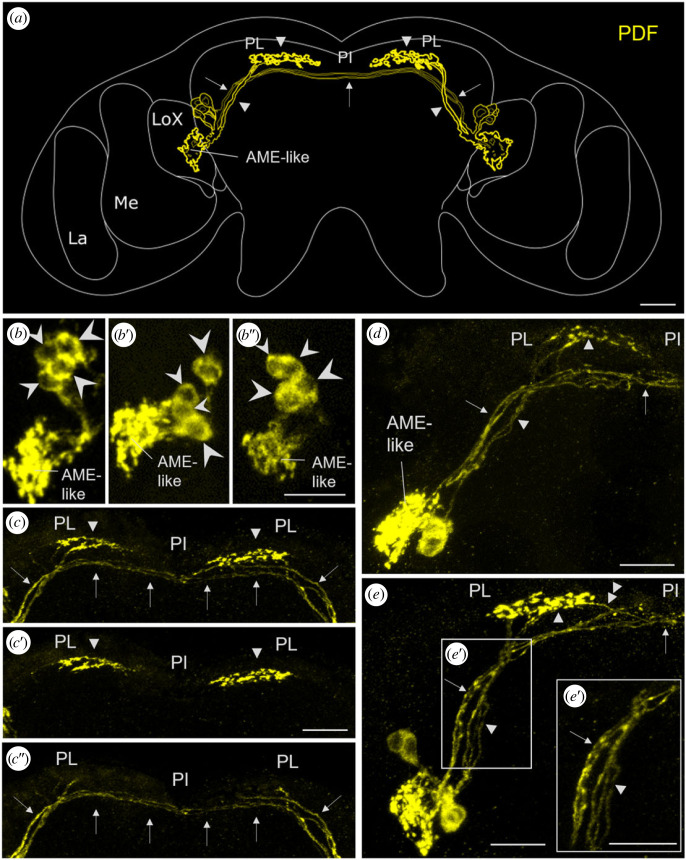
PDF-like immunostaining in the pea aphid brain. (*a*) Schematic representation of the PDF-ir neurons and their neurites. All four neurons appear to send neurites into an AME-like structure. Two main fibre bundles run medially; one crosses in the *pars intercerebralis* (PI) to the contralateral hemisphere and connects both AME-like structures with each other (arrows), the other runs to the *pars lateralis* (PL), where it shows varicose terminals (arrowheads). LA: lamina; ME: medulla; LoX: lobula complex. (*b*) Somata of the PDF-ir neurons from the left brain hemispheres of three different aphid brains. Their slightly different soma size is indicated by differently sized arrowheads. (*c*) PDF-ir fibres in the superior protocerebrum. Arrows mark the fibres projecting to the contralateral AME-like and arrowheads mark the fibres ending in the ipsilateral PL. (*c*) Full Z stack (20 stacks, thickness 1.5 µm) of the PDF fibres in the superior protocerebrum. (*c*’) Overlay of six more dorsally located confocal stacks showing the PDF-ir terminals in the PL. (c’’) Overlay of seven more ventrally located confocal stacks showing the contralateral PDF-ir fibres. (*d*) Left brain hemisphere showing that two fibres leaving the PDF-ir somata run to the PL and terminate there (arrowheads) and two others cross via the PI to the contralateral brain hemisphere. (*e*) Example of a brain hemisphere, in which three fibres run to the PL and one of them continues toward the PI. (*e*’) Magnification of the area in the rectangle of (*e*). Scale bars: 20 µm.

Using the newly generated antibody against 21 amino acids of the *A. pisum* PDF precursor (figure 1*b*) on brain whole-mounts, we found four PDF-immunoreactive (PDF-ir) cell bodies per hemisphere, located between the central brain and the optic lobe (figure 2*a*,*b*). These neurons slightly differed in size. Two of the four PDF-ir neurons possessed large cell bodies (mean area at the confocal plane showing the maximal size of the cell = 57.4 µm^2^ ± 1.7 µm^2^ s.e., *n* = 12), the other two were clearly smaller (mean area: 37 µm^2^ ± 1.3 µm^2^ s.e., *n* = 9) (figure 2*b*). Sometimes the two smaller cells had somata of similar size, but often we could distinguish one of intermediate size and one of a rather small size (figure 2*b*’,*b*’’). The somata of the four cells were always very close together and the neurites originating from them largely intermingled with each other, so that it was impossible to follow the neurites from individual neurons. Nevertheless, we were able to count the number of fibres in certain fibre tracts. The neurites of all four neurons appeared to innervate a neuropil located anterior to the lobula complex and proximally to the medulla that strongly resembled the accessory medulla (AME) of other insects [49–51] (figure 2*a*,*b*,*d*,*e*). In the following, we will call this structure AME-like region. We never saw any neurites running beyond the AME-like structure and entering the optic lobes or the compound eyes (figure 2*a*). At least two neurons from each hemisphere projected to the contralateral AME-like region, respectively (arrow in figure 2*a*,*c*,*d*,*e*’) and two neurons sent fibres to the *pars lateralis* in the superior protocerebrum, where they terminated by forming prominent varicosities (arrowheads in figure 2*a*,*c*,*d*). The fibres forming the commissure to the contralateral brain hemisphere were clearly distinguishable from the fibres terminating in the *pars lateralis*, because they were in different depths of the brain (figure 2*c*,*c*’,*c*’’). Sometimes a single fibre among those terminating in the *pars intercerebralis* appeared to depart from the varicosities and join the bundle of fibres projecting to the opposite hemisphere (double arrowhead in figure 2*e*).

### 2.4. The pigment-dispersing factor-immunoreactive cells are Cryptochrome-negative but period-positive clock neurons

**Figure 3.**
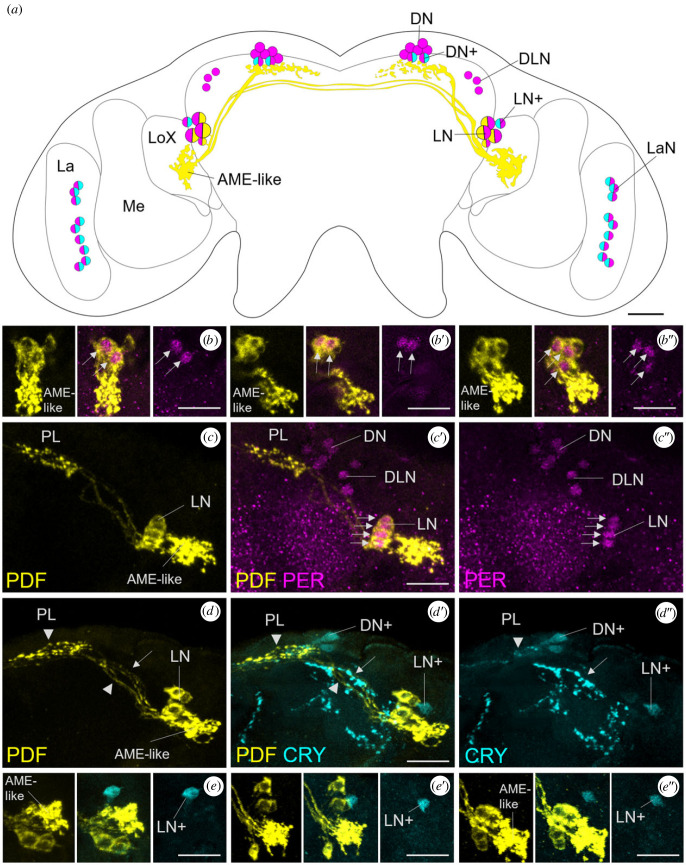
PDF and PER are colocalized in the CRY-negative lateral clock neurons (LN) of the pea aphid brain. (*a*) Schematic representation of the PDF-ir (yellow), PER-ir (magenta) and CRY-ir (cyan) neurons. DN: dorsal neurons; DN+: CRY-positive dorsal neurons; DLN: dorsolateral neurons; LN: lateral neurons; LN+: CRY-positive lateral neuron; LaN: lamina neurons; La: lamina; Me: medulla; LoX: lobula complex; AME-like: accessory medulla like structure. (*b*,*b*’’) Overlays of three–five confocal stacks showing PDF and PER double-labelling in the LNs of three brains stained at ZT23. Arrows mark the double-labelled cells (two LNs in *b* and *b*’ and three LNs in *b*’’). (*c*,*c*’’) Overlay of 21 confocal stacks showing PDF and PER double-labelling in the right brain hemisphere at ZT11. All four PDF-ir neurons express also PER (arrows). (*d*,*d*’’) Overlay of 10 confocal stacks depicting PDF and CRY double-labelling in the right brain hemisphere at ZT23. The CRY-positive LN+ is PDF-negative, but the projections of the LN+ overlap with the PDF-positive fibres projecting contralaterally (arrow). In addition, the fibres stemming from the CRY-positive DN + s overlap with the PDF-positive terminals in the *pars lateralis* (PL) (arrowhead). (*e*,*e*’’) Overlays of three–five confocal stacks showing PDF and CRY labelling in the LNs and LN+ of three brains. The CRY-positive LN+ was always PDF negative. Scale bars: 20 µm.

To test whether the PDF-ir cells are clock neurons, we performed co-labelling of anti-PDF with anti-Period (PER) and anti-CRY. Aphids were collected in the early morning, 1 h before lights-on (ZT23), and in the afternoon, 6 h before lights-off (ZT11). At these times (especially at ZT11), our previous study has detected significant PER staining in the lateral clock neurons (two CRY-negative LNs and one CRY-positive LN+), in three dorsolateral clock neurons (DLNs) and approximately seven dorsal clock neurons (seven CRY-negative DNs and two CRY-positive DN + s) and approximately nine lamina neurons (LaNs) [30]. Besides the one LN + and the two DN + s, CRY was present in all LaNs (figure 3*a*) [30]. Since we did not find PDF in the optic lobes, we did not further consider PER and CRY staining in the lamina in the present study.

Consistent with our previous study, we found PER in the nuclei of the LNs, DLNs and DNs (figure 3). At ZT23, PER staining was identical to the previous description and found in the three lateral neurons (two LNs and one LN+). PDF was present in the cytoplasm of the two PER-positive LNs (figure 3*b*). According to the size of the somata, these two PER/PDF-positive cells corresponded to the large PDF-ir neurons (figure 3*b*). In rare cases, we found a third PER/PDF-positive cell at ZT23 (figure 3*b*’’). At ZT11, PER staining was stronger and usually present in the nuclei of all four PDF-positive neurons (figure 3*c*). Co-staining with anti-PDF and anti-CRY showed that PDF and CRY never co-localized in the same neurons, although the neurites of the PDF and CRY-positive cells partly overlapped (figure 3*d*,*e*).

We conclude that all four PDF-ir neurons are PER-positive clock neurons that do not express CRY. In our previous study, we had obviously overlooked two of the LNs due to low PER staining intensity.

### 2.5. The pigment-dispersing factor-immunoreactive fibres overlap with fibres stemming from the Cryptochrome-positive clock neurons (LN+ and two DNs)

Although the CRY-positive LN+ was PDF-negative, the fibres arising from it always overlapped with the PDF-positive fibres that projected contralaterally (arrow in figure 3*d*). Furthermore, the fibres stemming from the two CRY-positive DN + s overlapped with the PDF terminals in the *pars lateralis* (arrowhead in figure 3*d*). This suggests that the CRY-positive and CRY-negative clock neurons communicate with each other.

### 2.6. The pigment-dispersing factor-immunoreactive terminals in the *pars lateralis* overlap with dendrites from the insulin-like peptide-producing neurosecretory cells

**Figure 4.**
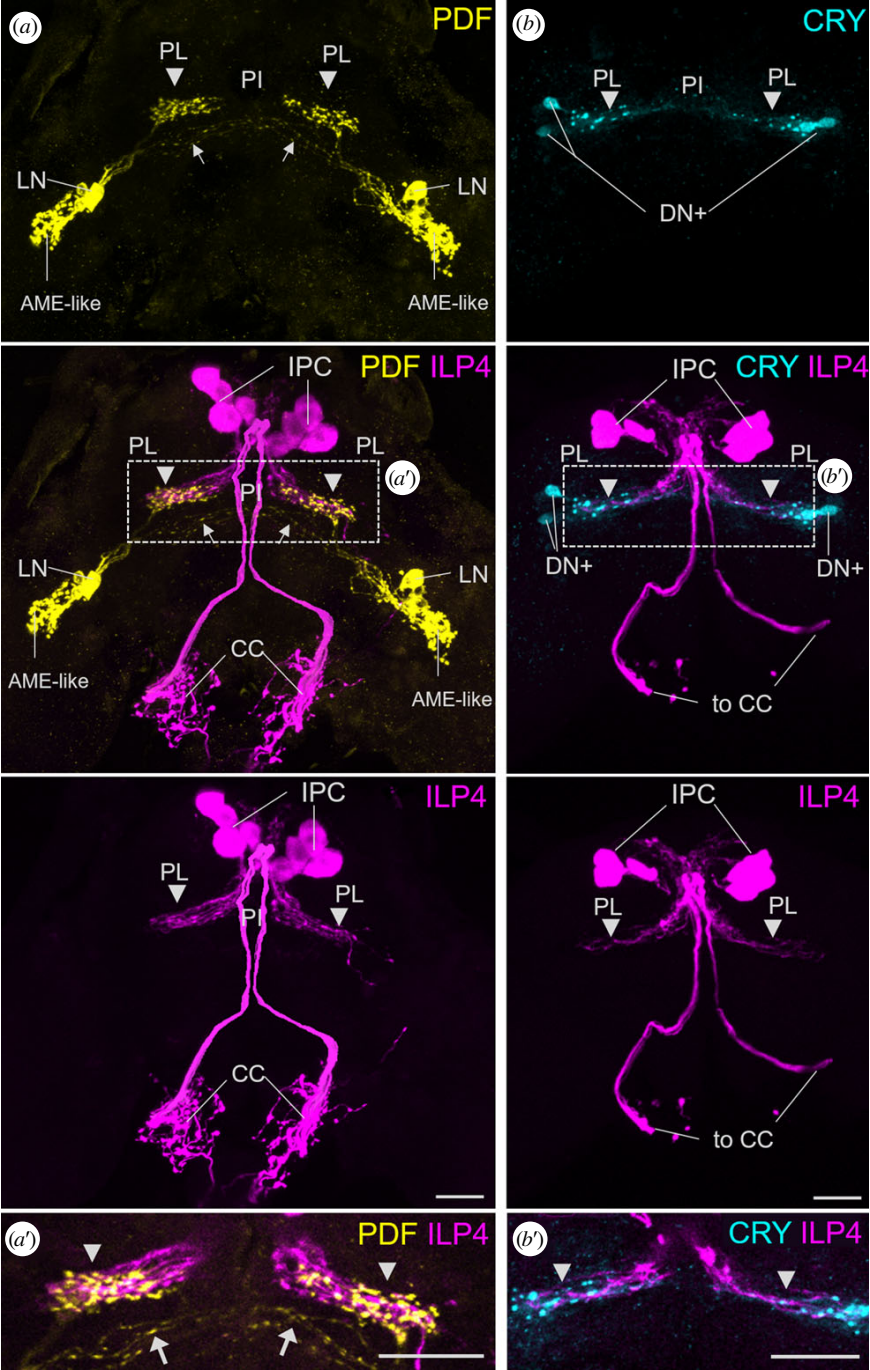
The PDF terminals of the LN and the fibres from the CRY-positive DN + s overlap with putative dendrites of the insulin-like peptide-producing cells (IPCs) in the *pars lateralis* (PL). (*a*) Overlay of 50 confocal stacks depicting PDF-ir and ILP4-ir in the brain and corpora cardiaca (CC). The ILP4-positive IPCs have dendrites in the PL and send axons to the CC from where they release ILP4 into the circulation. In the PL, their dendrites overlap with the PDF terminals from the PDF-positive LNs (arrowheads). The arrows mark the PDF fibres running contralaterally. (*a*’) A single confocal stack (2 µm thickness) showing the overlay of the PDF- and ILP4-positive fibres in the area indicated by the rectangle in (*a*). (*b*) Overlay of 30 confocal stacks showing CRY-ir and ILP4-ir in the brain and the projections to the corpora cardiaca (to CC). Only the CRY-positive dorsal clock neurons (DN+) are shown that project to the *pars intercerebralis* (PI) and overlap with the dendrites of the IPCs in the PL. (*b*’) A single confocal stack (2 µm thickness) showing the overlay of the CRY- and ILP4-positive fibres in the area indicated by the rectangle in (*b*). Labelling as in figure 3. Scale bars: 20 µm.

Our previous study has shown that insulin-like peptide 4 (ILP4) is a promising candidate for being the predicted virginoparin responsible for the switch between parthenogenesis and sexual reproduction in aphids [52]. The ILP4-producing neurosecretory cells (IPCs) in the *pars intercerebralis* have putative dendritic connections to the *pars lateralis* suggesting a possible communication between the circadian and photoperiodic systems. To elaborate this further, we performed double-immunolabelling with anti-PDF and anti-ILP4 as well as with anti-CRY and anti-ILP4. We found that the putative ILP4 dendrites fully overlap with the PDF terminals in the *pars lateralis* (figure 4*a*,*a*’) as well as with the CRY-positive fibres arising from the DN + s (figure 4*b*,*b*’).

### 2.7. *Pdf* expression shows daily and seasonal differences in abundance

**Figure 5.**
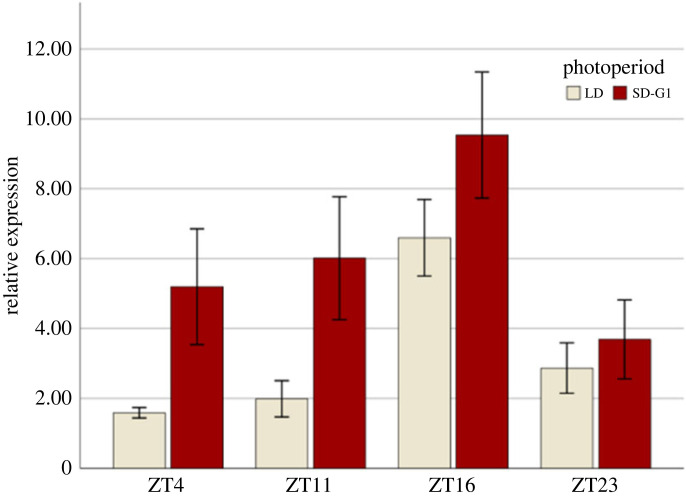
Results from RT-qPCR assays comparing the expression of the PDF-coding gene in two photoperiods and four timepoints along the day in *A. pisum*. Relative expression levels of the pea aphid PDF-coding gene at four timepoints and two photoperiod conditions (see Material and methods). Bars are mean values of three replicates ± s.e.m.

Since PDF has been shown to be involved in daily rhythms as well as in photoperiodism in other insects (see Introduction), and pea aphids show diurnal feeding rhythms and are paradigmatic photoperiodic insects, we investigated whether *Pdf* gene expression was affected by the time of day and day length. We compared the expression of the *Pdf* gene in head extracts at four different times of the day in two groups of aphids: aphids reared under LD (summer-like) and aphids that had been under SDs since they were embryos (see Material and methods) (figure 5). We found that *Pdf* gene expression was highly dependent on the time of day (two-way ANOVA: *F*_3,16_ = 7.70; *p* = 0.002) with the highest expression at ZT16 (on average 2.6 times higher than at other ZTs). Furthermore, *Pdf* expression depended significantly on day length (two-way ANOVA: *F*_1,16_ = 11.02; *p* = 0.004). On average, the *Pdf-*encoding gene showed double the expression (2.04 times) under SD than under LD (figure 5). No significant interaction effects were observed between photoperiod and time of day: *F*_3,16_ = 0.994; *p* = 0.421. We conclude that *Pdf* expression shows daily oscillations and that SDs strongly induce *Pdf* expression.

### 2.8. The length of the pigment-dispersing factor-immunoreactive terminals in the *pars lateralis* varies on a daily basis

**Figure 6.**
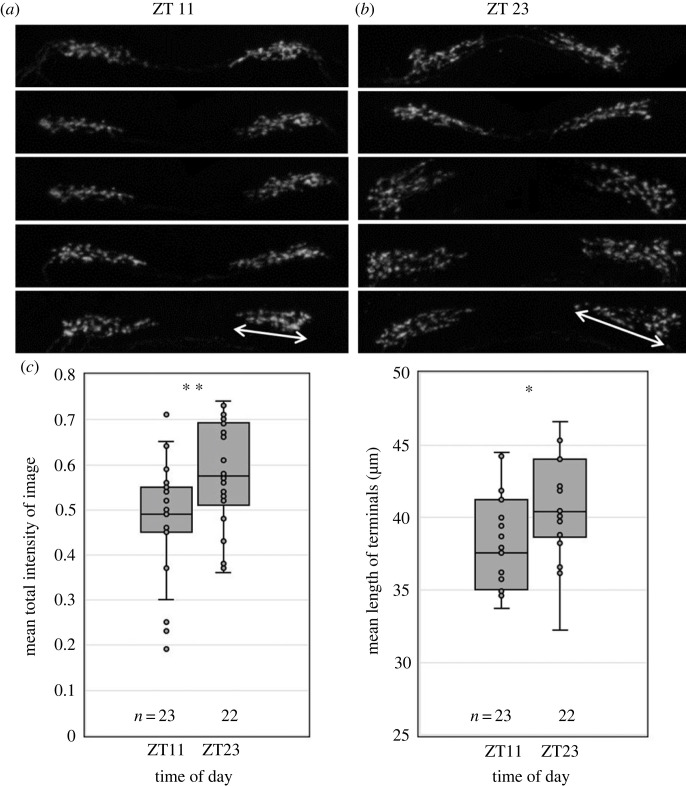
The PDF terminals in the *pars lateralis* are plastic and change their daily appearance. (*a*,*b*) Appearance of the PDF terminals in five representative brains at Zeitgeber time (ZT) 11 and ZT23, respectively, under LD conditions. Each image represents an overlay of 10–15 confocal stacks. (*c*) Mean staining intensity of all images containing the PDF terminals (i) and mean length of the terminals (ii) at ZT11 and ZT23, respectively. The number (*n*) of quantified brains is indicated below the boxplots. Staining intensity was significantly different between ZT11 and ZT23 (two-sample *t*-test; *p* = 0.006). The same was true for terminal length (Kruskal–Wallis test; *p* = 0.01). * *p* < 0.05; ** *p* < 0.01.

After we found that *Pdf* expression shows diurnal oscillations, we aimed to investigate whether the PDF peptide might be used as a circadian clock signal. Therefore, we tested whether the PDF-staining intensity, under LDs (i.e. 16 h photoperiod), varies during the day and the PDF terminals change their daily shape as was observed in *D. melanogaster* [53,54]. Assuming that PDF peptide abundance peaks after the peak in *Pdf* gene expression, we stained the aphid brains at the expected PDF trough in the early morning, 1 h before lights-on (ZT23), and at its expected maximum in the afternoon, 6 h before lights-off (ZT11). We used the method of Hermann-Luibl *et al*. [55], who determined the pixel intensity within a defined area that contained the entire terminals (figure 6*a*,*b*). We found that the mean staining intensity in this area was significantly higher at ZT23 than at ZT11 (figure 6*c*i). To confirm the difference between ZTs, we additionally measured the length of the terminals as indicated by ‘double-headed’ arrows in figure 6*a*,*b* and found that they are significantly longer at ZT23 than at ZT11 (figure 6*c*ii). We conclude that the PDF terminals are plastic and change their shape throughout the day. This makes them suited to transfer daily signals of the lateral clock neurons to downstream neurons.

### 2.9. The pigment-dispersing factor-immunoreactive terminals are longer under short days than under long days

**Figure 7.**
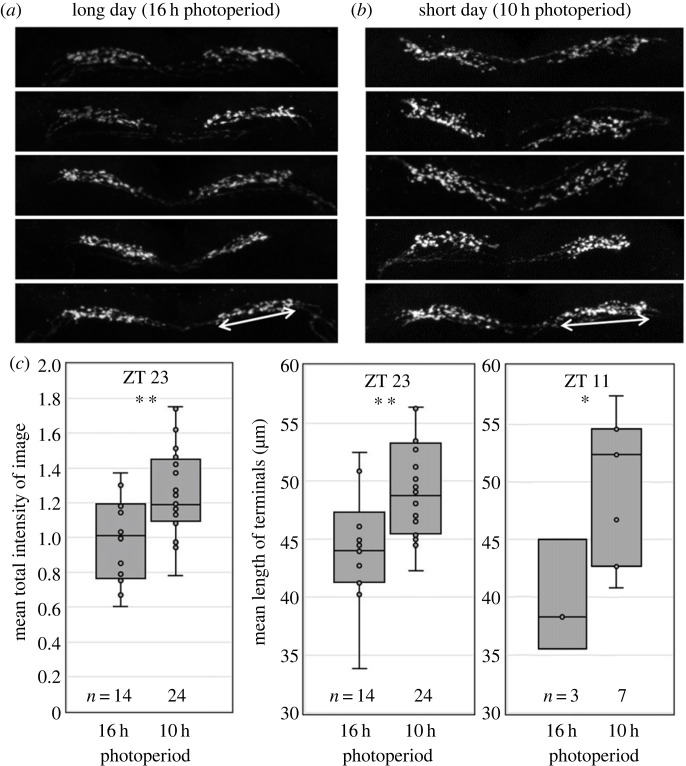
The PDF terminals in the *pars lateralis* are longer under SDs than under LDs. (*a*,*b*) Appearance of the PDF terminals in five representative brains entrained to long and short photoperiods, respectively, at ZT23. (*c*) Mean staining intensity of all images containing the PDF terminals stained at ZT23 (i) and mean length of the terminals at ZT23 (ii) and ZT11 (iii) under LD (16 h) and SD (10 h), respectively. The number of quantified brains (*n*) is indicated below the boxplots. Staining intensity at ZT23 was significantly different between long (16 h) and short (10 h) photoperiods (two-sample *t*-test; *p* = 0.004). The same was true for terminal length at ZT23 and ZT11 (two-sample *t*-test; *p* = 0.004 and 0.041, respectively). * *p* < 0.05; ** *p* < 0.01.

To test whether the PDF terminals change their size when aphids experience SDs, we immunostained the brains of animals maintained under LDs and of aphids that were reared under SDs. We stained most brains at ZT23, because we had found more prominent PDF terminals at that time, but we performed also some immunostainings at ZT11. We found that the PDF terminals significantly increased in size under SDs as compared to LDs, and this was true at ZT23 and ZT11, although the number of brains stained at ZT11 was too low to exclude effects of the time of day completely (figure 7). In order to investigate the effects of time of day on PDF-immunostaining under LDs and SDs, complete time-course experiments should be carried out in the future. Under SDs, the PDF fibres spread toward the *pars intercerebralis*, and sometimes the fibres stemming from the two brain hemispheres even touched each other (figure 7*b*). This suggests that in aphids, PDF signalling increases under SDs when the aphid starts to produce sexual morphs, and that PDF might be the clock factor of aphids communicating day length to the IPCs.

## 3. Discussion

### 3.1. A divergent *pdf* gene evolved in the Aphidomorpha

The pea aphid, *Acyrthosiphon pisum*, belonged to the few insects in which the *pdf* gene and PDF peptide were not identified. Although the absence of PDF could explain the apparently weak circadian rhythmicity of aphids [31,56], it remained questionable as to why this important peptide, which is present in virtually all panarthropods [9,57–60], should be absent in the strongly photoperiodic aphids. When the *A. pisum* neuropeptide genes were first analysed [29], the PDF gene was not recognized as such because of the significant differences with the other then known insect PDF precursors. A comparative analysis including other aphid and aphid-related species now allowed us to identify the *pdf* gene. The aphid PDF neuropeptide may lack the 7 C-terminal amino acids that are typical for PDF [42], but its N-terminal sequence is well conserved [60] and its expression pattern in the brain shows large similarities to that of other insect species (see discussion below). There is thus little doubt that this is indeed the aphid PDF gene.

However, differently from other insects [9], the aphid PDF cannot be C-terminally amidated, which might drastically affect its physiological function, as α-amidation is thought to be essential for biological activity of many neuropeptides [61]. It has been argued that amidation greatly affects the binding affinity of peptides to their G-protein-coupled receptors [62,63], but our current data do not allow us to ascertain if this binding is modified in aphids. Indeed, a gene coding for a PDF receptor (PDFR) is predicted in aphids [37] and the analysis of the predicted protein sequence shows that there is no evidence of major differences between the aphid sequence and that of other insects. Although a few highly conserved positions in other insects are different in the predicted PDFR of *A. pisum*, the highly conserved motifs and binding domains described for family B of GPCRs [64,65] are also present in the *A. pisum* sequence (data not shown). However, identification of the mature aphid PDF neuropeptide would be needed to test it as a ligand of the predicted PDFR. It has also been speculated that α-amidation would have a role in protecting peptides from enzymatic degradation (thus increasing their half-lifes) when released into circulation [61,66]. However, we have no evidence that PDF is released into hemolymph, and thus the loss of the C-terminal amide would not compromise the integrity of aphid PDF and it would not have been selected against.

Aphids may be subject to high selective pressures because, as rather static plant suckers, they are particularly exposed to changing environmental conditions and they are special in several aspects. For example, aphids have a rather high number of visual and non-visual pigments that may help them to perceive light, avoid light and be protected from light [67–70]. Most relevant, core clock genes *period* (*per*) and *timeless* (*tim*) experimented high evolutionary rates [71]. Interestingly, these genes participate in the circadian clock feedback loop involved in light perception. Furthermore, aphids have lost the protein Jetlag, which is involved in the synchronization of the clock with the daily light–dark cycle [71]. However, this rapid evolution is not a general trend of all aphid clock genes since genes involved in the other feedback loop (i.e. *Clock* and *cycle*) evolved at expected rates [71]. We may speculate that whatever is the selection pressure that drove the divergent evolution of core clock proteins PER and TIM it might have also similarly driven the evolution of the divergent clock neuropeptide PDF. The fact that the levels of *period* gene transcription, similar to the PDF-coding gene, are significantly influenced by the photoperiod (with SDs inducing higher expression of both genes) speaks for this hypothesis [69,71]. Thus, it is possible that the function of particular clock genes (including PDF) has been directed towards photoperiod-related tasks at the expense of circadian clock ones. Alternatively, relaxed selective constraints may have led to a divergent PDF in the ancestor of all Aphidomorpha (electronic supplementary material, figure S1). However, its extreme conservation in all aphid lineages through their *ca* 200 Myr of evolution points to a strong purifying selection operating to preserve its functionality.

### 3.2. The pigment-dispersing factor-positive clock neurons in the aphid brain closely resemble those of other insects

The expression pattern of PDF in aphid circadian clock neurons strongly resembles that of other insects [72–75]. As true for flies, cockroaches, bugs and bees [18,73,76–78], PDF is present in aphid lateral clock neurons with different soma sizes. These clock neurons send projections to the superior protocerebrum and to the contralateral brain hemisphere, more precisely to a neuropil that strongly resembles the AME of other insects. We could not distinguish the projection patterns of the different neurons, but it is most likely that those with larger somata project to the contralateral and those with smaller somata remain in the ipsilateral brain hemisphere as was found in cockroaches and flies [50,79]. The AME was first identified as a circadian pacemaker centre in hemimetabolous insects [80–82] and was later established as clock centre in most insects (reviewed in [83]). In these insects, it serves as communication centre for circadian clock neurons and receives photoreceptor input from the compound eyes and extraretinal photoreceptors.

Despite all similarities to other insects, the PDF neurons of aphids are special in the sense that they completely lack PDF fibres in the optic lobes, which would argue in favour of a functional divergence. Interestingly, the coleopteran species *Holotrichia parallela* is the only other known insect, as documented by Hamanaka *et al*. [84], that also lacks PDF fibres in the optic lobe.

Furthermore, the AME is particularly rich in varicosities, which are store and release sites of neuropeptides. This speaks against a prominent role of the AME, or at least the PDF fibres in the AME, as a light-input pathway to the circadian clock neurons. Most interestingly, a rather sparse innervation of the optic lobes by PDF fibres was also found in honeybees [51,78]. Furthermore, many varicose endings and no fine dendritic-like fibres were found in the AME of honeybee larvae. This has been interpreted as a lack of photoreceptor input into the honeybee AME, which may be explained by the different lifestyle of bees, which rely much more on social cues than eye-transmitted light–dark cycles to synchronize their circadian clocks [85–87].

Similarly, compound eyes do not appear to play a role as photoreceptors for photoperiodic responses in aphids [88], which may explain the absence of dendritic PDF fibres in AME. Instead, the photoperiodic photoreceptor of aphids was localized to the lateral superior protocerebrum [88], where CRY1-positive clock neurons were later found [30,69]. There, the fibres of these CRY1-positive clock neurons intermingle with fibres of the PDF neurons making it likely that the photosensitive CRY1 not only synchronizes the circadian clock of aphids but at the same time transfers information about day length (photoperiod) to the PDF-positive clock neurons.

Since aphids have a damped circadian clock [31,56], it is possible that PDF plays a weaker role in circadian rhythmicity in aphids compared with other insects. Perhaps the main function of PDF in aphids is the promotion of winter diapause (which in aphids takes the form of sexual reproduction; see Introduction) [4,89,90].

### 3.3. Pigment-dispersing factor as putative factor promoting aphid sexual reproduction in autumn

While both the photoperiodic photoreceptors and the photoperiodic timer appear to localize in the lateral superior protocerebrum (*pars lateralis*) [91], whether the aphid produces sexual or asexual progeny is controlled by neurosecretory cells in the median superior protocerebrum (*pars intercerebralis*) [91]. Ablation of these neurosecretory cells led to the production of sexual morphs even under LDs suggesting that they produce a parthenogenesis promoting substance, also called virginoparin [91,92]. Later studies suggested that ILPs are the virginoparin in question [52,93]. ILP1 and ILP4 are produced in four neurosecretory cells in the *pars intercerebralis* of each brain hemisphere and their expression significantly diminishes under SDs promoting sexual reproduction [52]. These neurons project to the corpora cardiaca and from there three nerves (two laterals and one medial) go to the abdomen where ILPs might be released close to the developing aphid embryos, and their levels determine their fate either as parthenogenetic females or as sexual morphs.

**Figure 8.**
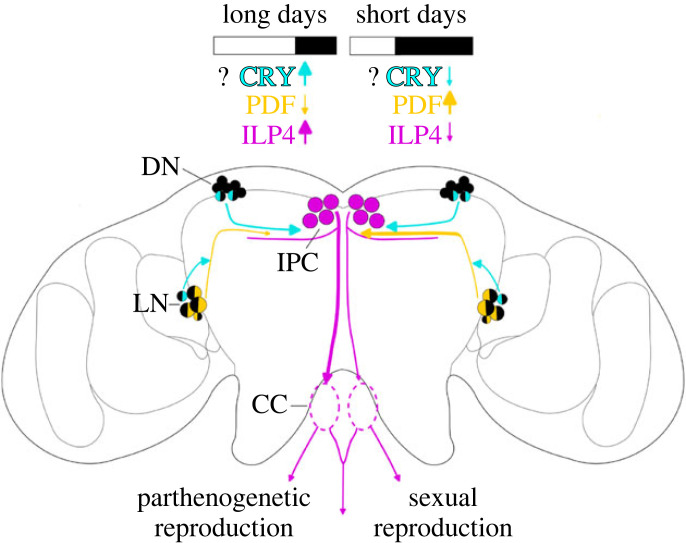
Possible mechanisms of PDF, CRY and ILP4 signalling during LDs and SDs. On LDs (left hemisphere), PDF signalling from the lateral clock neurons (LN) is low, as indicated by the short PDF terminals contacting insulin-producing cells (IPC) in the pars intercerebralis, while ILP4 signalling is high [50]. ILP4 signalling to aphid gonads ensures parthenogenetic reproduction. On SDs (right hemisphere), PDF signalling increases, as seen in the long PDF terminals. Consequently, ILP4 signalling is reduced, allowing a switch to sexual reproduction. CRY is expressed in one LN and two dorsal clock (DN) neurons and may function as a seasonal photoreceptor that signals more strongly during LDs than SDs, but this remains to be experimentally demonstrated.

Here, we show that the dendrites of the ILP4 expressing neurosecretory cells extend toward the *pars lateralis* where they overlap with the terminals of PDF-positive as well as CRY-positive clock neurons. This strongly suggests that this is the region where the information about photoperiod and time-of-day is transferred to these neurosecretory cells. Furthermore, we show that *pdf* expression is significantly higher and that the PDF terminals extend further towards the *pars intercerebralis* under short photoperiods as compared to long photoperiods. Thus, PDF signalling to the ILP neurons may be stronger under short photoperiods and this may in turn result in a decrease of ILP signalling and promote the development of embryos as sexual morphs (figure 8 summarizes this scenario). Further experiments, double staining PDF and ILP, in both LD and SD aphids, would be necessary to confirm this hypothesis. A similar role of PDF in communicating short photoperiods to the photoperiodic system has been shown for the bugs *Plautia stali* [18] and *Pyrrhocoris apterus* [19], while very recently, Hidalgo *et al*. [20] showed that PDF is the LD-signalling factor in *Drosophila melanogaster*. In *D. melanogaster*, the PDF terminals are very prominent under LDs, activate the ILP-producing cells in the *pars intercerebralis* and prevent the flies from going into dormancy. Under SDs, the PDF terminals become less prominent so that the dormancy inducing factor *Eyes Absent* in the ILP-producing cells can become active and induce dormancy.

In summary, we show here that PDF levels could be part of the signal communicating the photoperiod to the *pars intercerebralis* also in aphids. Put in a simple way, PDF may control the synthesis of ILPs to determine the fate of the developing embryos either as parthenogenetic females or as sexual morphs. Further experiments are necessary to prove the function of PDF in the seasonal control of aphid reproduction, but our present results set the stage for future studies in this direction.

## 4. Material and methods

### 4.1. Aphid strains and rearing

*Acyrthosiphon pisum* aphids of the LSR1 strain were used for most experiments in the present report. LSR1 is the pea aphid strain whose genome was firstly sequenced [46]. This strain has been maintained in our laboratory on *Vicia fabae* seedlings for more than 8 years under LD photoperiod conditions (i.e. 16 h lights-on and 8 h of darkness, or 16L : 8D) at 18°C. Strain LSR1 produces sexual females and males when reared under SD conditions (i.e. less than or equal to 12 h lights-on and greater than or equal to 12 h of darkness). For experimental validation of PDF sequences, additional pea aphid strains were used in addition to LSR1 (see strain details in the electronic supplementary material, table S1).

### 4.2. Identification of pigment-dispersing factor-encoding genes in aphid genomes

The PDF sequence as predicted from the *Rhodnius prolixus* genome with the surrounding convertase cleavage sites (KRNSEIINSLLGIPKVLIDAGR, obtained using a tblastn search on the *R. prolixus* genome with the *Drosophila* PDF sequence as a query) was used as query in a tblastn search on the *A. pisum* NCBI Genome Reference Sequence database (Annotation Release 103, June 2019). If the putative *A. pisum* PDF sequence found would represent a functional neuropeptide, it should be conserved and expressed in other aphid species. It was therefore used as a query in a tblastn search of the Aphidomorpha transcriptome shotgun assemblies available at NCBI.

Additional Aphidomorpha PDF homologues were obtained using BlastP searches against the NCBI Protein Reference Sequence database or against the Aphid Genome Database at the BioInformatics Platform for Agroecosystem Arthropods (BIPAA, INRAE, France). We also performed tblastn searches against whole aphid genome or transcriptome sequences at NCBI. In this latter case, sequences were translated before their inclusion in the alignment (electronic supplementary material, table S2, provides accession numbers and details of these searches). Alignment of aphid sequences (including the predicted *A. pisum* sequence) was done using ClustalX 2.0 [94]. Phylogenetic reconstructions and calculations of number of amino acid differences between aphid PDF sequences were conducted using MEGA version 11 [95].

### 4.3. Experimental validation of the pea aphid pigment-dispersing factor-encoding gene

To experimentally validate gene models, total RNA was extracted from groups of four–five aphids of the above-described strains using TRI Reagent® (T9424, Sigma-Aldrich, USA) and Direct-Zol RNA extraction Kit (R2052, Zymo Research, USA) following suppliers' recommendations. RNA was quantified by spectrophotometry using a NanoDrop ND-1000 (Nanodrop Technologies, Inc., Wilmington, DE, USA) and stored at −80°C until use. Total RNA (1–5 µg) was used for cDNA synthesis using the NZY First-strand cDNA Synthesis Kit (MB40001, NZYTech, Portugal), primed with a mix of oligo (dT)18 to enrich the samples with cDNA from mature mRNAs and hexamers. Primers to PCR amplify the predicted aphid PDF gene transcript on cDNA were designed based on 5′ and 3′ UTRs of the predicted transcript model. Forward primer was T1-F1 (TCATAATCACCGAAGACAGCAAG) and R0 (CAGTTTGTATGTGCGTTACCTACG) was the reverse primer (figure 1). Amplified products were directly sequenced using PCR primers after purification through 4 M ammonium acetate–ethanol precipitation. Direct sequencing was done using the ABI Prism BigDye® terminator v3.1 Cycle Sequencing kit (Applied Biosystems) in an ABI3730XL sequencer. Chromatogram handling and processing was performed using the STADEN package [96]. To resolve allelic combinations present at two observed polymorphic positions, we proceeded to clone the amplified fragments for three of the strains sequenced (strains LSR1, SUT and BOL; see electronic supplementary material, table S1). For cloning the amplified fragments, we used the NZY-A PCR cloning kit and NZYStar Competent Cells (MB053 and MB00501, NZYTech, Portugal). Inserts contained in recombinant plasmids were sequenced as described above using plasmid-based primers T7 (TAATACGACTCACTATAGGG) and M13 (GTTTTCCCAGTCACGACGT).

### 4.4. Quantification of *pdf* gene expression by RT-qPCR

Excised aphid heads from aphids of the LSR1 strain were used to compare the expression of the PDF gene under two photoperiodic conditions and at four different timepoints along the day. Synchronized adult aphids reared under LD and under SD conditions were sampled the following day after their final moult at ZTs 4, 11, 16 and 23. Aphid samples were kept at −80°C until RNA extraction. LD aphids had been reared under a 16L : 8D regime while SD aphids were under 10L : 14D conditions. For SD aphids, the G1 generation was used [71]. Three groups of five aphids were used as replicates for each condition. Total RNA was extracted from heads from the frozen aphid samples and quantified as described above. For cDNA synthesis, we used the Superscript III kit (Invitrogen) on 1 µg (*ca*) of total RNA primed with oligo (dT)18 and random hexamers following supplier's recommendations.

RT-qPCR was performed using an AriaMx Real-Time PCR System (Agilent) and SYBR qPCR Master Mix (HY-K0501, Med Chem Express, Sweden). Primers used for RT-qPCR were QF4 (ATCCGTTGCGTACTACCTATTG) and QR4 (CATCTTCCACGTGTCTCTTACC). For each sample, three technical replicates were done. The RpL7 gene was used as an endogenous control of constitutive expression [97]. Efficiencies of the PDF and of the RpL7 primer pairs were 103.7 and 90.8%, respectively. Relative expression for each sample was calculated using the ΔΔCt (threshold cycle) method [98]. All relative expression values were normalized to an inter-run calibrator sample consisting of a cDNA synthesized from a mix of total aphid RNAs obtained from whole insects at different developmental stages. Two-way ANOVA was used to analyse the effects of photoperiod and ZT on gene expression with SPSS Statistics 28.0 software [99].

### 4.5. Aphid pigment-dispersing factor antiserum and specificity assay

A polyclonal antiserum against a synthetic peptide (CSLYVPDDNFVIEEQNAPIAT) corresponding to a region of the *A. pisum* PAP (figure 1) was raised in guinea pigs by Moravian-Biotechnology Ltd (Brno, Czech Republic). We opted to make an antiserum to a part of the PDF precursor rather than the neuropeptide itself as it allowed for a longer peptide sequence as antigen thereby potentially increasing our chances of obtaining a good antiserum; obviously PDF and its precursor are always produced together and, as described for other neuropeptides, at least part of the precursor proteins are expected to be retained along with the neuropeptides in the Golgi secretory vesicles, and thus immunohistochemical visualization of precursor peptides should be reflective of the localization of the neuropeptide [100,101]. The synthetic peptide was custom synthesized by Shanghai RoyoBiotech Co. Ltd (Shanghai, China, 201200). After immunization of a guinea pig and animal bleeding antisera were obtained by affinity purification column using the synthetic peptide used as immunogen. Small neuropeptide antisera may cross-react with other neuropeptides and polyclonal antisera may even recognize entirely different epitopes. We therefore chose to have the antiserum purified on an affinity column made with the same peptide and therefore the final serum contains only antibodies that recognize the synthetic peptide used as antigen.

### 4.6. Brain dissections and immunohistochemistry

Adult aphids, reared as described in §4.1, were collected on the second or third day after their final moult. For the comparison between LD and SD conditions, we reared synchronized aphids until the third nymphal stage and then we split them in two groups. One remained under LD conditions, while the other was transferred to SD conditions (10L : 14D). The next generation of aphids was collected when they became adults. Adults were fixed in 4% paraformaldehyde in PBST (phosphate-buffered saline, PBS, containing 0.5% Triton-X100) for 4 h at room temperature (RT). They were washed 3 × 10 min in PBS and then the brains were dissected in PBS. Brains were incubated in NGS solution (5% normal goat serum in PBST) for 2 h at RT or overnight at 4°C.

For the immunostaining against PDF and the co-immunostainings against PDF and ILP4 or PDF and CRY, we applied the following protocol. Brains were incubated in the primary antibody solution (NGS 5%, NaN_3_ 0.02% and primary antibodies in PBST) for 2 days. The following antibody dilutions were used: PDF 1 : 1000, PDF-CRY 1 : 5000–1 : 1000, respectively, and PDF-ILP4 1 : 5000–1 : 5000, respectively. Brains were then washed 6 × 10 min in PBST and incubated in secondary antibody solution (5% normal goat serum in PBST, Alexa Fluor 488 or 633 goat anti-guinea pig 1 : 200; Alexa Fluor 488 goat anti-rat 1 : 200; Alexa Fluor 555 or 633 goat anti-rabbit 1 : 200 (Thermo Scientific)) for 4 h at RT, then washed 4 × 10 min in PBST and 1 × 10 min in PBS. Brains were then put on specimen slides and embedded in Vectashield Antifade mounting medium (Vector Laboratories, Burlingame, CA). Slides were stored at 4°C until scanning. For the co-immunostaining against PER and PDF, we applied the primary antibodies sequentially, because the PDF staining was very strong and appeared to interfere with the PER staining. First, we incubated the brains for two days at 4°C in PER primary antibody solution (NGS 5%, NaN_3_ 0.02%, PER 1 : 2000 in PBST) and followed the same procedure described above until the application of the secondary antibody solution (5% normal goat serum in PBST, Alexa Fluor 488 anti-guinea pig 1 : 200 (Thermo Scientific)). Subsequently, we washed the brains 6 × 10 min with PBST and incubated them for 1 day at RT with the PDF primary antibody solution (NGS 5%, NaN_3_ 0.02%, PDF 1 : 5000 in PBST). Then, brains were washed 6 × 10 min in PBST, incubated in secondary antibody solution for 4 h at RT (5% normal goat serum in PBST, Alexa Fluor 633 anti-guinea pig 1 : 200 (Thermo Scientific)) and finally washed 4 × 10 min in PBST and 1 × 10 min in PBS. Brains were then put on specimen slides and embedded in Vectashield Antifade mounting medium (Vector Laboratories, Burlingame, CA). Slides were stored at 4°C until scanning. Electronic supplementary material, table S3, provides details on all the antibodies used.

### 4.7. Microscopy and imaging

All the immunostainings apart from the PDF-PER double-labelling were visualized with a Leica TCS SPE confocal microscope (Leica, Wetzlar, Germany). We used a 20-fold or 40-fold glycerol immersion objective (ACS APO Leica Microsystem, Wetzlar, Germany), and the confocal images were acquired with a resolution of 1024 × 1024 pixels and z-axis intervals of 2 µm.

For the PDF-PER double-labelling, we used a Leica CLSM SP8 (Leica Microsystems, Wetzlar, Germany). We used a 20-fold glycerol immersion objective (HC PL APO, Leica Microsystem, Wetzlar, Germany) and similarly to before, the confocal images were acquired with a resolution of 1024 × 1024 pixels and z-axis intervals of 2 µm.

The confocal stacks were analysed with Fiji ImageJ [102]. Only contrast, brightness, background correction and colour scheme adjustments were applied to the confocal images.

### 4.8. Quantification of pigment-dispersing factor

For PDF quantification in the *pars lateralis* terminals, samples were processed in exactly the same way during the staining protocol and were scanned with identical laser settings. In order to compare the aphids raised in LDs (16L : 8D) and SDs (10L : 14D), we collected the insects at the same moment from the entraining chambers and performed the staining procedure simultaneously. To quantify the intensity of the PDF terminals in the *pars lateralis* we used the method described in [55]. For each brain, we compiled maximum projections (encompassing 10–15 confocal stacks), which contained the PDF terminals in the *pars lateralis* of both brain hemispheres. Images were then cut to 100 000 pixels (500 pixels wide and 200 pixels high; see figures 4 and 6) taking care that the entire PDF terminals were in the image. All resulting images were therefore of the exact same size and contained only the PDF terminals in the *pars lateralis*. We then set the background of each image to zero and measured the mean total intensity of the whole image, which reflected the extension and intensity of the dorsal projection terminals. These manipulations were done without knowing the ZT or the photoperiod at which the samples were taken to avoid any subjective influence of the investigator.

To measure the length of the terminals on the compiled maximum projections (see figures 4 and 6), we traced a line spanning through the terminals and measuring its length (Command: Analyse → Measure) in ImageJ. For most brains, we measured the length of both PDF terminals and then calculated a mean length out of the two values. When a hemisphere was damaged or the PDF terminals were too curved, we restricted our measurements to the intact hemisphere.

Two-sample *t*-test (for normally distributed data) or Kruskal–Wallis test (for not normally distributed data) were used to test for significant differences in PDF intensity and terminal length. The statistical tests were performed in R v. 4.2.2 [103].

## Data Availability

Sequences obtained in this work have been deposited in GenBank with accession numbers indicated in the electronic supplementary material, table S1 [104].
